# 2-(4-Chloro­phen­yl)acetic acid–2-{(*E*)-[(*E*)-2-(2-pyridyl­methyl­idene)hydrazin-1-yl­idene]meth­yl}pyridine (1/1)

**DOI:** 10.1107/S1600536810032721

**Published:** 2010-08-21

**Authors:** Hadi D. Arman, Trupta Kaulgud, Edward R. T. Tiekink

**Affiliations:** a Department of Chemistry, The University of Texas at San Antonio, One UTSA Circle, San Antonio, Texas 78249-0698, USA; b Department of Chemistry, University of Malaya, 50603 Kuala Lumpur, Malaysia

## Abstract

In the crystal of the title 1:1 adduct, C_8_H_7_ClO_2_·C_12_H_10_N_4_, the components are linked by an O—H⋯N hydrogen bond between the carb­oxy­lic acid and one of the pyridine N atoms. In the acid, the carb­oxy­lic acid group is approximately normal to [dihedral angle = 72.9 (2)°] but twisted with respect to the plane through the benzene ring [C—C—C—O torsion angle = 25.4 (5)°]. The base is roughly planar [dihedral angle between rings = 12.66 (15)°; r.m.s. deviation of the 16 non-H atoms = 0.107 Å] and the conformations about both imine bonds are *E*. The dimeric aggregates are linked into a supra­molecular layer in the *ab* plane by C—H⋯O inter­actions.

## Related literature

For related studies on co-crystal formation, see: Broker & Tiekink (2007 ▶); Broker *et al.* (2008 ▶); Arman *et al.* (2010 ▶).
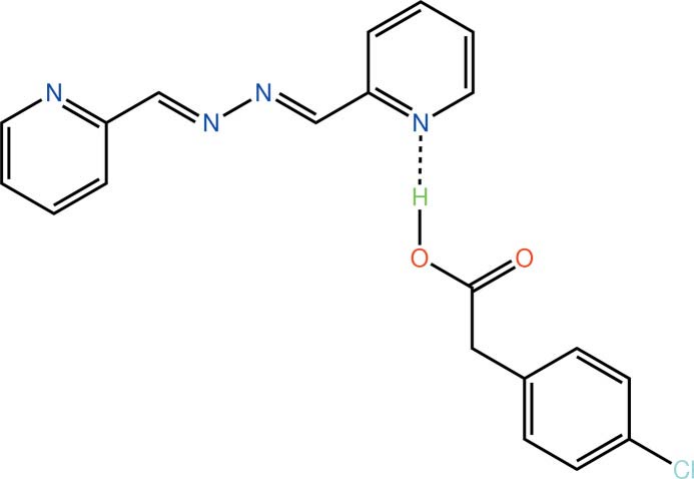


## Experimental

### 

#### Crystal data

C_8_H_7_ClO_2_·C_12_H_10_N_4_
*M* *_r_* = 380.83
Orthorhombic, 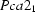

*a* = 11.740 (6) Å
*b* = 4.641 (2) Å
*c* = 33.451 (15) Å
*V* = 1822.6 (15) Å^3^
*Z* = 4
Mo *K*α radiation
μ = 0.23 mm^−1^
*T* = 98 K
0.22 × 0.19 × 0.09 mm

#### Data collection

Rigaku Saturn724 diffractometer
Absorption correction: multi-scan (*ABSCOR*; Higashi, 1995 ▶) *T*_min_ = 0.625, *T*_max_ = 1.000
7554 measured reflections
4091 independent reflections
3661 reflections with *I* > 2σ(*I*)
*R*_int_ = 0.046

#### Refinement

*R*[*F* ^2^ > 2σ(*F* ^2^)] = 0.057
*wR*(*F* ^2^) = 0.113
*S* = 1.14
4091 reflections
247 parameters
2 restraints
H atoms treated by a mixture of independent and constrained refinement
Δρ_max_ = 0.28 e Å^−3^
Δρ_min_ = −0.24 e Å^−3^
Absolute structure: Flack (1983 ▶), 1973 Friedel pairs
Flack parameter: 0.09 (8)

### 

Data collection: *CrystalClear* (Molecular Structure Corporation & Rigaku, 2005 ▶); cell refinement: *CrystalClear*; data reduction: *CrystalClear*; program(s) used to solve structure: *SHELXS97* (Sheldrick, 2008 ▶); program(s) used to refine structure: *SHELXL97* (Sheldrick, 2008 ▶); molecular graphics: *ORTEP-3* (Farrugia, 1997 ▶) and *DIAMOND* (Brandenburg, 2006 ▶); software used to prepare material for publication: *publCIF* (Westrip, 2010 ▶).

## Supplementary Material

Crystal structure: contains datablocks global, I. DOI: 10.1107/S1600536810032721/hb5608sup1.cif

Structure factors: contains datablocks I. DOI: 10.1107/S1600536810032721/hb5608Isup2.hkl

Additional supplementary materials:  crystallographic information; 3D view; checkCIF report

## Figures and Tables

**Table 1:** Hydrogen-bond geometry (Å, °)

*D*—H⋯*A*	*D*—H	H⋯*A*	*D*⋯*A*	*D*—H⋯*A*
O1—H1*o*⋯N1	0.85 (3)	1.89 (3)	2.734 (4)	173 (3)
C9—H9⋯O2	0.95	2.51	3.215 (4)	131
C10—H10⋯O1^i^	0.95	2.57	3.493 (4)	164

Symmetry code: (i) 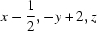.

**Fig. 1.**
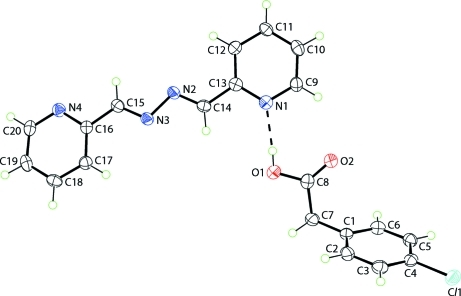
Molecular structure of the constituents of co-crystal (I) showing displacement ellipsoids at the 50% probability level. The O–H···N hydrogen bond is shown as a dashed line.

**Fig. 2.**
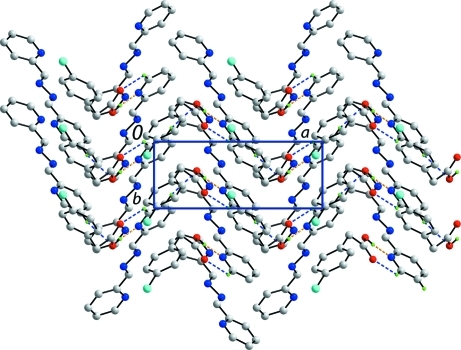
Supramolecular layer formation in the ab plane in (I). Dimeric aggregates, connected by an O–H···N hydrogen bond (orange dashed lines) are connected via C–H···O contacts (blue dashed lines). Hydrogen atoms not involved in the aforementioned interactions have been omitted for reasons of clarity.

**Fig. 3.**
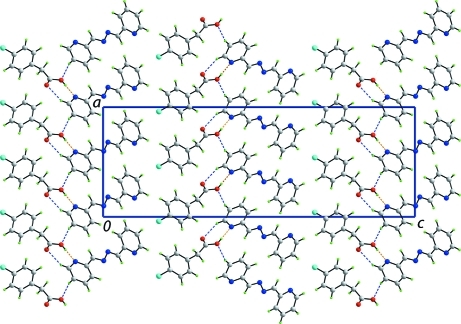
Stacking of layers along the c axis in (I). The O–H···N (orange) and C–H···O (blue) contacts are shown as dashed lines.

## supplementary crystallographic information

### Comment

Co-crystallization of carboxylic acids with pyridine-containing bases has led to several structural motifs as well as salts (Broker & Tiekink, 2007; Broker *et al.*, 2008; Arman *et al.*, 2010). In continuation of these studies, the co-crystallization experiment between 2-(4-chlorophenyl)acetic acid and 2-[(1*E*)-[(*E*)-2-(pyridin-2-ylmethylidene)hydrazin-1-ylidene] methyl]pyridine in a 1:1 ratio in their methanol solution was investigated. This lead to the isolation of the title 1:1 co-crystal, (I).

The constituents of (I), Fig. 1, are connected by a O–H···N hydrogen bond where the N is a pyridine-N, rather than an imine-N, as usually seen in co-crystals of this type (Broker *et al.*, 2008), Table 1. In the acid, the dihedral angle formed between the carboxylic acid group and the benzene ring is 72.9 (2) ° and the former is twisted with respect to the plane of the benzene ring as seen in the value of the C1–C7–C8–O2 torsion angle of 25.4 (5) °. In the base, the 16 non-hydrogen atoms are almost co-planar with the r.m.s. deviation being 0.107 Å [max. deviations are 0.132 (3) Å for atom C18 and -0.153 (2) Å for the N1 atom]. The greatest twist in the base is found about the C15–C16 bond as seen in the N3–C15–C16–C17 torsion angle of 6.0 (5) °. The pyridine-N atoms are *anti* with respect to each other and the conformation about each imine bond [N2═C14 = 1.276 (4) Å and N3═C15 = 1.280 (4) Å] is *E*, again observations normally seen in related systems (Broker *et al.*, 2008).

In the crystal packing, the dimeric aggregates held together by the O–H···N hydrogen bonds are linked into a supramolecular array in the *ab* plane *via* C–H···O contacts, Fig. 2 and Table 1. The resulting layers stack along the *c* direction, Fig. 3.

### Experimental

Yellow crystals of (I) were isolated from the 1/1 co-crystallization of 2-[(1*E*)-[(*E*)-2-(pyridin-2-ylmethylidene)hydrazin-1-ylidene]methyl]pyridine (Sigma-Aldrich; 0.095 mmol) and 2-(4-chlorophenyl)acetic acid (Sigma-Aldrich; 0.094 mmol) in a methanol solution, m. pt. 370 - 373 K.

### Refinement

C-bound H-atoms were placed in calculated positions (C–H 0.95–0.99 Å) and were included in the refinement in the riding model approximation with *U*_iso_(H) set to 1.2*U*_eq_(C). The O-bound H-atom was located in a difference Fourier map and was refined with a distance restraint of O–H = 0.84±0.01 Å, and with *U*_iso_(H) = 1.5*U*_eq_(O).

### Crystal data

**Table:**

C_8_H_7_ClO_2_·C_12_H_10_N_4_	*F*(000) = 792
*M**_r_* = 380.83	*D*_x_ = 1.388 Mg m^−^^3^
Orthorhombic, *P**c**a*2_1_	Mo *K*α radiation, λ = 0.71073 Å
Hall symbol: P 2c -2ac	Cell parameters from 4996 reflections
*a* = 11.740 (6) Å	θ = 2.1–40.3°
*b* = 4.641 (2) Å	µ = 0.23 mm^−^^1^
*c* = 33.451 (15) Å	*T* = 98 K
*V* = 1822.6 (15) Å^3^	Prism, yellow
*Z* = 4	0.22 × 0.19 × 0.09 mm

### Data collection

**Table:**

Rigaku Saturn724 diffractometer	4091 independent reflections
Radiation source: sealed tube	3661 reflections with *I* > 2σ(*I*)
graphite	*R*_int_ = 0.046
Detector resolution: 28.5714 pixels mm^-1^	θ_max_ = 27.5°, θ_min_ = 2.4°
ω scans	*h* = −4→15
Absorption correction: multi-scan (*ABSCOR*; Higashi, 1995)	*k* = −4→6
*T*_min_ = 0.625, *T*_max_ = 1.000	*l* = −43→42
7554 measured reflections	

### Refinement

**Table:**

Refinement on *F*^2^	Secondary atom site location: difference Fourier map
Least-squares matrix: full	Hydrogen site location: inferred from neighbouring sites
*R*[*F*^2^ > 2σ(*F*^2^)] = 0.057	H atoms treated by a mixture of independent and constrained refinement
*wR*(*F*^2^) = 0.113	*w* = 1/[σ^2^(*F*_o_^2^) + (0.0265*P*)^2^ + 0.6335*P*] where *P* = (*F*_o_^2^ + 2*F*_c_^2^)/3
*S* = 1.14	(Δ/σ)_max_ = 0.001
4091 reflections	Δρ_max_ = 0.28 e Å^−^^3^
247 parameters	Δρ_min_ = −0.24 e Å^−^^3^
2 restraints	Absolute structure: Flack (1983), 1973 Friedel pairs
Primary atom site location: structure-invariant direct methods	Flack parameter: 0.09 (8)

### Special details

**Table:**

Geometry. All s.u.'s (except the s.u. in the dihedral angle between two l.s. planes) are estimated using the full covariance matrix. The cell s.u.'s are taken into account individually in the estimation of s.u.'s in distances, angles and torsion angles; correlations between s.u.'s in cell parameters are only used when they are defined by crystal symmetry. An approximate (isotropic) treatment of cell s.u.'s is used for estimating s.u.'s involving l.s. planes.
Refinement. Refinement of *F*^2^ against ALL reflections. The weighted *R*-factor *wR* and goodness of fit *S* are based on *F*^2^, conventional *R*-factors *R* are based on *F*, with *F* set to zero for negative *F*^2^. The threshold expression of *F*^2^ > 2σ(*F*^2^) is used only for calculating *R*-factors(gt) *etc*. and is not relevant to the choice of reflections for refinement. *R*-factors based on *F*^2^ are statistically about twice as large as those based on *F*, and *R*- factors based on ALL data will be even larger.

### Fractional atomic coordinates and isotropic or equivalent isotropic displacement parameters (Å^2^)

**Table:**

	*x*	*y*	*z*	*U*_iso_*/*U*_eq_	
Cl1	0.54028 (7)	1.20255 (16)	0.67877 (3)	0.03461 (19)	
O1	0.2462 (2)	0.4385 (5)	0.86536 (7)	0.0339 (6)	
H1o	0.201 (3)	0.542 (7)	0.8788 (10)	0.051*	
O2	0.2010 (2)	0.7655 (5)	0.81878 (7)	0.0349 (6)	
N1	0.0916 (2)	0.7320 (5)	0.91096 (8)	0.0273 (6)	
N2	0.0996 (2)	0.3341 (5)	1.00194 (8)	0.0256 (6)	
N3	0.1807 (2)	0.1275 (5)	1.01538 (8)	0.0249 (6)	
N4	0.1708 (2)	−0.3295 (6)	1.10056 (8)	0.0278 (6)	
C1	0.3916 (3)	0.5966 (7)	0.77127 (10)	0.0273 (7)	
C2	0.4965 (3)	0.7262 (7)	0.77713 (10)	0.0311 (7)	
H2	0.5380	0.6864	0.8009	0.037*	
C3	0.5427 (3)	0.9140 (7)	0.74890 (10)	0.0308 (7)	
H3	0.6146	1.0022	0.7533	0.037*	
C4	0.4823 (3)	0.9690 (6)	0.71456 (10)	0.0261 (7)	
C5	0.3768 (3)	0.8454 (7)	0.70749 (10)	0.0291 (7)	
H5	0.3354	0.8872	0.6838	0.035*	
C6	0.3332 (3)	0.6583 (7)	0.73615 (10)	0.0297 (7)	
H6	0.2613	0.5701	0.7316	0.036*	
C7	0.3417 (3)	0.4012 (7)	0.80335 (10)	0.0319 (8)	
H7A	0.3044	0.2341	0.7904	0.038*	
H7B	0.4040	0.3272	0.8204	0.038*	
C8	0.2559 (3)	0.5567 (7)	0.82911 (10)	0.0276 (7)	
C9	0.0329 (3)	0.9348 (7)	0.89091 (10)	0.0284 (7)	
H9	0.0593	0.9934	0.8653	0.034*	
C10	−0.0646 (3)	1.0608 (7)	0.90619 (10)	0.0302 (7)	
H10	−0.1038	1.2045	0.8914	0.036*	
C11	−0.1048 (3)	0.9744 (7)	0.94373 (10)	0.0263 (7)	
H11	−0.1721	1.0564	0.9547	0.032*	
C12	−0.0443 (3)	0.7666 (6)	0.96461 (10)	0.0255 (6)	
H12	−0.0696	0.7024	0.9901	0.031*	
C13	0.0548 (3)	0.6533 (6)	0.94746 (10)	0.0245 (6)	
C14	0.1254 (3)	0.4354 (6)	0.96765 (9)	0.0252 (7)	
H14	0.1924	0.3680	0.9547	0.030*	
C15	0.1501 (3)	0.0090 (6)	1.04822 (9)	0.0250 (7)	
H15	0.0799	0.0642	1.0601	0.030*	
C16	0.2202 (3)	−0.2089 (6)	1.06803 (9)	0.0220 (6)	
C17	0.3292 (3)	−0.2836 (6)	1.05470 (10)	0.0265 (7)	
H17	0.3609	−0.1966	1.0315	0.032*	
C18	0.3902 (3)	−0.4885 (7)	1.07626 (10)	0.0287 (7)	
H18	0.4648	−0.5416	1.0682	0.034*	
C19	0.3413 (3)	−0.6131 (7)	1.10929 (10)	0.0306 (7)	
H19	0.3815	−0.7537	1.1244	0.037*	
C20	0.2316 (3)	−0.5292 (7)	1.12030 (10)	0.0299 (7)	
H20	0.1980	−0.6182	1.1430	0.036*	

### Atomic displacement parameters (Å^2^)

**Table:**

	*U*^11^	*U*^22^	*U*^33^	*U*^12^	*U*^13^	*U*^23^
Cl1	0.0384 (4)	0.0313 (4)	0.0342 (4)	−0.0011 (3)	0.0090 (4)	0.0029 (4)
O1	0.0357 (14)	0.0361 (13)	0.0299 (14)	0.0045 (10)	0.0061 (10)	0.0038 (10)
O2	0.0357 (14)	0.0371 (13)	0.0320 (14)	0.0084 (11)	0.0047 (11)	0.0067 (11)
N1	0.0269 (14)	0.0267 (13)	0.0281 (14)	−0.0008 (11)	−0.0003 (11)	−0.0001 (12)
N2	0.0238 (13)	0.0274 (13)	0.0255 (14)	0.0036 (10)	−0.0027 (11)	0.0004 (11)
N3	0.0215 (13)	0.0276 (13)	0.0256 (14)	0.0015 (11)	−0.0025 (10)	−0.0002 (11)
N4	0.0273 (14)	0.0276 (14)	0.0285 (15)	−0.0005 (12)	−0.0004 (12)	0.0022 (11)
C1	0.0304 (17)	0.0263 (16)	0.0254 (16)	0.0081 (13)	0.0056 (13)	0.0002 (13)
C2	0.0298 (17)	0.0371 (18)	0.0263 (17)	0.0060 (14)	−0.0028 (13)	0.0000 (14)
C3	0.0238 (16)	0.0304 (16)	0.038 (2)	−0.0023 (13)	0.0010 (14)	−0.0014 (15)
C4	0.0313 (16)	0.0210 (14)	0.0259 (16)	0.0018 (13)	0.0058 (14)	−0.0032 (12)
C5	0.0296 (17)	0.0312 (16)	0.0266 (17)	0.0036 (13)	−0.0031 (13)	−0.0024 (14)
C6	0.0268 (16)	0.0287 (16)	0.0337 (19)	−0.0007 (13)	0.0005 (14)	−0.0039 (14)
C7	0.0338 (18)	0.0256 (16)	0.036 (2)	0.0058 (14)	0.0059 (15)	0.0021 (14)
C8	0.0264 (16)	0.0275 (16)	0.0288 (17)	−0.0079 (13)	−0.0023 (14)	0.0020 (14)
C9	0.0296 (17)	0.0298 (17)	0.0256 (17)	0.0004 (13)	−0.0025 (13)	0.0038 (14)
C10	0.0327 (17)	0.0265 (15)	0.0313 (19)	0.0019 (14)	−0.0106 (15)	0.0006 (14)
C11	0.0220 (16)	0.0257 (15)	0.0314 (17)	0.0007 (12)	−0.0035 (13)	0.0005 (14)
C12	0.0244 (15)	0.0250 (15)	0.0270 (16)	−0.0056 (12)	0.0015 (13)	0.0003 (13)
C13	0.0235 (15)	0.0226 (14)	0.0273 (17)	−0.0033 (12)	−0.0025 (13)	−0.0032 (12)
C14	0.0212 (15)	0.0249 (16)	0.0294 (17)	−0.0006 (12)	−0.0001 (13)	−0.0020 (13)
C15	0.0244 (16)	0.0244 (15)	0.0263 (16)	0.0006 (12)	−0.0038 (13)	−0.0026 (12)
C16	0.0243 (15)	0.0193 (13)	0.0223 (15)	−0.0027 (12)	−0.0019 (12)	−0.0013 (12)
C17	0.0253 (17)	0.0273 (16)	0.0269 (17)	−0.0021 (13)	0.0002 (13)	0.0018 (14)
C18	0.0193 (15)	0.0299 (16)	0.037 (2)	−0.0005 (13)	−0.0029 (13)	−0.0037 (14)
C19	0.0378 (18)	0.0256 (16)	0.0283 (18)	0.0004 (14)	−0.0123 (14)	0.0019 (14)
C20	0.0383 (19)	0.0292 (16)	0.0222 (16)	−0.0014 (14)	−0.0031 (14)	0.0020 (13)

### Geometric parameters (Å, °)

**Table:**

Cl1—C4	1.753 (3)	C7—H7A	0.9900
O1—C8	1.336 (4)	C7—H7B	0.9900
O1—H1o	0.85 (3)	C9—C10	1.383 (5)
O2—C8	1.214 (4)	C9—H9	0.9500
N1—C13	1.346 (4)	C10—C11	1.400 (5)
N1—C9	1.346 (4)	C10—H10	0.9500
N2—C14	1.276 (4)	C11—C12	1.387 (4)
N2—N3	1.424 (3)	C11—H11	0.9500
N3—C15	1.280 (4)	C12—C13	1.399 (4)
N4—C20	1.343 (4)	C12—H12	0.9500
N4—C16	1.354 (4)	C13—C14	1.472 (4)
C1—C2	1.385 (5)	C14—H14	0.9500
C1—C6	1.391 (5)	C15—C16	1.463 (4)
C1—C7	1.522 (4)	C15—H15	0.9500
C2—C3	1.394 (5)	C16—C17	1.399 (4)
C2—H2	0.9500	C17—C18	1.391 (4)
C3—C4	1.373 (5)	C17—H17	0.9500
C3—H3	0.9500	C18—C19	1.373 (5)
C4—C5	1.386 (4)	C18—H18	0.9500
C5—C6	1.391 (5)	C19—C20	1.396 (5)
C5—H5	0.9500	C19—H19	0.9500
C6—H6	0.9500	C20—H20	0.9500
C7—C8	1.509 (4)		
			
C8—O1—H1o	108 (3)	C10—C9—H9	118.8
C13—N1—C9	118.5 (3)	C9—C10—C11	119.3 (3)
C14—N2—N3	111.9 (3)	C9—C10—H10	120.4
C15—N3—N2	111.9 (3)	C11—C10—H10	120.4
C20—N4—C16	116.9 (3)	C12—C11—C10	118.6 (3)
C2—C1—C6	118.0 (3)	C12—C11—H11	120.7
C2—C1—C7	120.1 (3)	C10—C11—H11	120.7
C6—C1—C7	121.9 (3)	C11—C12—C13	118.7 (3)
C1—C2—C3	121.4 (3)	C11—C12—H12	120.6
C1—C2—H2	119.3	C13—C12—H12	120.6
C3—C2—H2	119.3	N1—C13—C12	122.5 (3)
C4—C3—C2	118.8 (3)	N1—C13—C14	115.0 (3)
C4—C3—H3	120.6	C12—C13—C14	122.5 (3)
C2—C3—H3	120.6	N2—C14—C13	122.1 (3)
C3—C4—C5	121.8 (3)	N2—C14—H14	118.9
C3—C4—Cl1	119.1 (2)	C13—C14—H14	118.9
C5—C4—Cl1	119.1 (3)	N3—C15—C16	121.9 (3)
C4—C5—C6	118.0 (3)	N3—C15—H15	119.1
C4—C5—H5	121.0	C16—C15—H15	119.1
C6—C5—H5	121.0	N4—C16—C17	123.1 (3)
C1—C6—C5	122.0 (3)	N4—C16—C15	114.1 (3)
C1—C6—H6	119.0	C17—C16—C15	122.8 (3)
C5—C6—H6	119.0	C18—C17—C16	118.3 (3)
C8—C7—C1	112.0 (3)	C18—C17—H17	120.8
C8—C7—H7A	109.2	C16—C17—H17	120.8
C1—C7—H7A	109.2	C19—C18—C17	119.4 (3)
C8—C7—H7B	109.2	C19—C18—H18	120.3
C1—C7—H7B	109.2	C17—C18—H18	120.3
H7A—C7—H7B	107.9	C18—C19—C20	118.7 (3)
O2—C8—O1	122.7 (3)	C18—C19—H19	120.6
O2—C8—C7	125.0 (3)	C20—C19—H19	120.6
O1—C8—C7	112.3 (3)	N4—C20—C19	123.6 (3)
N1—C9—C10	122.4 (3)	N4—C20—H20	118.2
N1—C9—H9	118.8	C19—C20—H20	118.2
			
C14—N2—N3—C15	−174.8 (3)	C9—N1—C13—C12	2.4 (5)
C6—C1—C2—C3	0.2 (5)	C9—N1—C13—C14	−178.6 (3)
C7—C1—C2—C3	−177.8 (3)	C11—C12—C13—N1	−2.1 (5)
C1—C2—C3—C4	−0.3 (5)	C11—C12—C13—C14	179.0 (3)
C2—C3—C4—C5	0.6 (5)	N3—N2—C14—C13	−179.8 (2)
C2—C3—C4—Cl1	−179.5 (2)	N1—C13—C14—N2	−178.1 (3)
C3—C4—C5—C6	−0.9 (5)	C12—C13—C14—N2	0.9 (5)
Cl1—C4—C5—C6	179.3 (2)	N2—N3—C15—C16	−179.6 (3)
C2—C1—C6—C5	−0.4 (5)	C20—N4—C16—C17	0.0 (4)
C7—C1—C6—C5	177.5 (3)	C20—N4—C16—C15	−179.3 (3)
C4—C5—C6—C1	0.7 (5)	N3—C15—C16—N4	−174.6 (3)
C2—C1—C7—C8	98.4 (4)	N3—C15—C16—C17	6.0 (5)
C6—C1—C7—C8	−79.5 (4)	N4—C16—C17—C18	−0.9 (5)
C1—C7—C8—O2	25.4 (5)	C15—C16—C17—C18	178.4 (3)
C1—C7—C8—O1	−154.6 (3)	C16—C17—C18—C19	0.9 (5)
C13—N1—C9—C10	−1.1 (5)	C17—C18—C19—C20	−0.1 (5)
N1—C9—C10—C11	−0.5 (5)	C16—N4—C20—C19	0.8 (5)
C9—C10—C11—C12	0.8 (5)	C18—C19—C20—N4	−0.8 (5)
C10—C11—C12—C13	0.4 (4)		

### Hydrogen-bond geometry (Å, °)

**Table:**

*D*—H···*A*	*D*—H	H···*A*	*D*···*A*	*D*—H···*A*
O1—H1o···N1	0.85 (3)	1.89 (3)	2.734 (4)	173 (3)
C9—H9···O2	0.95	2.51	3.215 (4)	131
C10—H10···O1^i^	0.95	2.57	3.493 (4)	164

Symmetry codes:  (i) *x*−1/2, −*y*+2, *z*.
